# Analysis of the response mechanisms of *Pinellia ternata* to terahertz wave stresses using transcriptome and metabolic data

**DOI:** 10.3389/fpls.2023.1227507

**Published:** 2023-09-12

**Authors:** Dongdong Wang, Surendra Sarsaiya, Xu Qian, Leilei Jin, Fuxing Shu, Chuanyou Zhang, Jishuang Chen

**Affiliations:** ^1^ College of Biotechnology and Pharmaceutical Engineering, Nanjing Tech University, Nanjing, Jiangsu, China; ^2^ Bioresource Institute for Healthy Utilization, Zunyi Medical University, Zunyi, Guizhou, China; ^3^ Nanjing Kuncan Technology Co., Nanjing, China

**Keywords:** *Pinellia ternata*, terahertz, transcriptome, metabonomic, plant hormones

## Abstract

*Pinellia ternata* (Thunb.) Breit. (Araceae), a significant medicinal plant, has been used to treat various diseases for centuries. Terahertz radiation (THZ) is located between microwaves and infrared rays on the electromagnetic spectrum. THZ possesses low single-photon energy and a spectral fingerprint, but its effects on plant growth have not yet been investigated. The study’s primary objective was to examine the transcriptome and metabolome databases of the SY line to provide a new perspective for identifying genes associated with resistance and growth promotion and comprehending the underlying molecular mechanism. Variations in the biological characteristics of *P. ternata* grown under control and experimental conditions were analyzed to determine the effect of THZ. Compared with the control group, phenotypic variables such as leaf length, petiole length, number of leaves, leaf petiole diameter, and proliferation coefficient exhibited significant differences. *P. ternata* response to THZ was analyzed regarding the effects of various coercions on root exudation. The experimental group contained considerably more sugar alcohol than the control group. The transcriptome analysis revealed 1,695 differentially expressed genes (DEGs), including 509 upregulated and 1,186 downregulated genes. In the KEGG-enriched plant hormone signaling pathway, there were 19 differentially expressed genes, 13 of which were downregulated and six of which were upregulated. In the metabolomic analysis, approximately 416 metabolites were uncovered. There were 112 DEMs that were downregulated, whereas 148 were upregulated. The *P. ternata* leaves displayed significant differences in phytohormone metabolites, specifically in brassinolide (BR) and abscisic acid (ABA). The rise in BR triggers alterations in internal plant hormones, resulting in faster growth and development of *P. ternata*. Our findings demonstrated a link between THZ and several metabolic pathway processes, which will enhance our understanding of *P. ternata* mechanisms.

## 1 Introduction

*Pinellia ternata* (Thunb.) Breit. (Araceae) is a traditional Chinese herb with a long history and thousands of known species. South Asia is the primary production region (Shu et al., 2021). The genetic material of wild *P. ternata* has a lengthy growth cycle and is highly susceptible to parasites and diseases due to the growing demand for *P. ternata* germplasm, which is essential to standardizing the production of *P. ternata* germplasm at a high throughput level. The new high-throughput plant bioreactor will be utilized to coculture *P. ternata* with the THZ energy ring, which is a source of electromagnetic and light waves and has the dual effect of electromagnetic and light waves and weak molecular interactions (Rezaei et al., 2022).

Terahertz waves (1 THz = 1012 Hz) are electromagnetic waves whose unique position in the electromagnetic spectrum makes them useful for material identification, medical imaging, and spectral analysis. Terahertz parametric oscillators based on stimulated electromagnetic coupler dispersion can generate THZ waves with strong directionality, a narrow pulse width, and high power at room temperature. It is the most prevalent method of producing THZ. Due to the rapid development of wave applications, such as immediate communication and surveillance technologies in the millimeter to THZ frequency ranges, the number of devices has increased in recent years (Zhan et al., 2023). As technology advances, researchers must investigate the biological effects of THZ radiation exposure. To our knowledge, however, a comprehensive assessment of the biological effects of electromagnetic radiation at these frequencies has yet to be conducted (Mattsson et al., 2018). Shirkavand et al. (2022) discovered that exposure to high-intensity THZ waves can generate heat.

Researchers have developed a novel technique for observing the morphology of THZ-irradiated cells. Due to the high-water absorption in the THZ region, preliminary investigations of the biological effects of THZ irradiation have exposed materials through the bottom of dishes (Wilmink and Grundt, 2011; Riccardo et al., 2022; Sasaki et al., 2022).

The THZ energy loop cocultured with *P. ternata* resulted in growth, possibly due to THZ waves accelerating water molecules in the culture solution, plant transport, or both. It has been demonstrated that the growth hormone affects plant growth and development by combining with the growth hormone response factor, auxin response factor (ARF), in the plant stress response pathway (Liu et al., 2022). Cytokinins are involved in several processes, such as plant cell division and differentiation, stem and root growth, maintenance of apical dominance, fruit and seed development, nutrient signaling, and responses to biotic and abiotic stresses (Li et al., 2021). The abscisic acid signaling pathway involves multiple microRNAs. The level of miR159 expression in Arabidopsis seeds is regulated by the concentration of abscisic acid and has a cleavage effect on the target gene transcription factors MYB101 and MYB33 (Reyes and Chua, 2007; Wang et al., 2021; Ali et al., 2022a; Ali et al., 2022b). Under thermal stress, putative genes for flavonoid biosynthesis have been studied in *P. ternata* (Raza et al., 2021; Guo et al., 2023).

**Figure 1:**
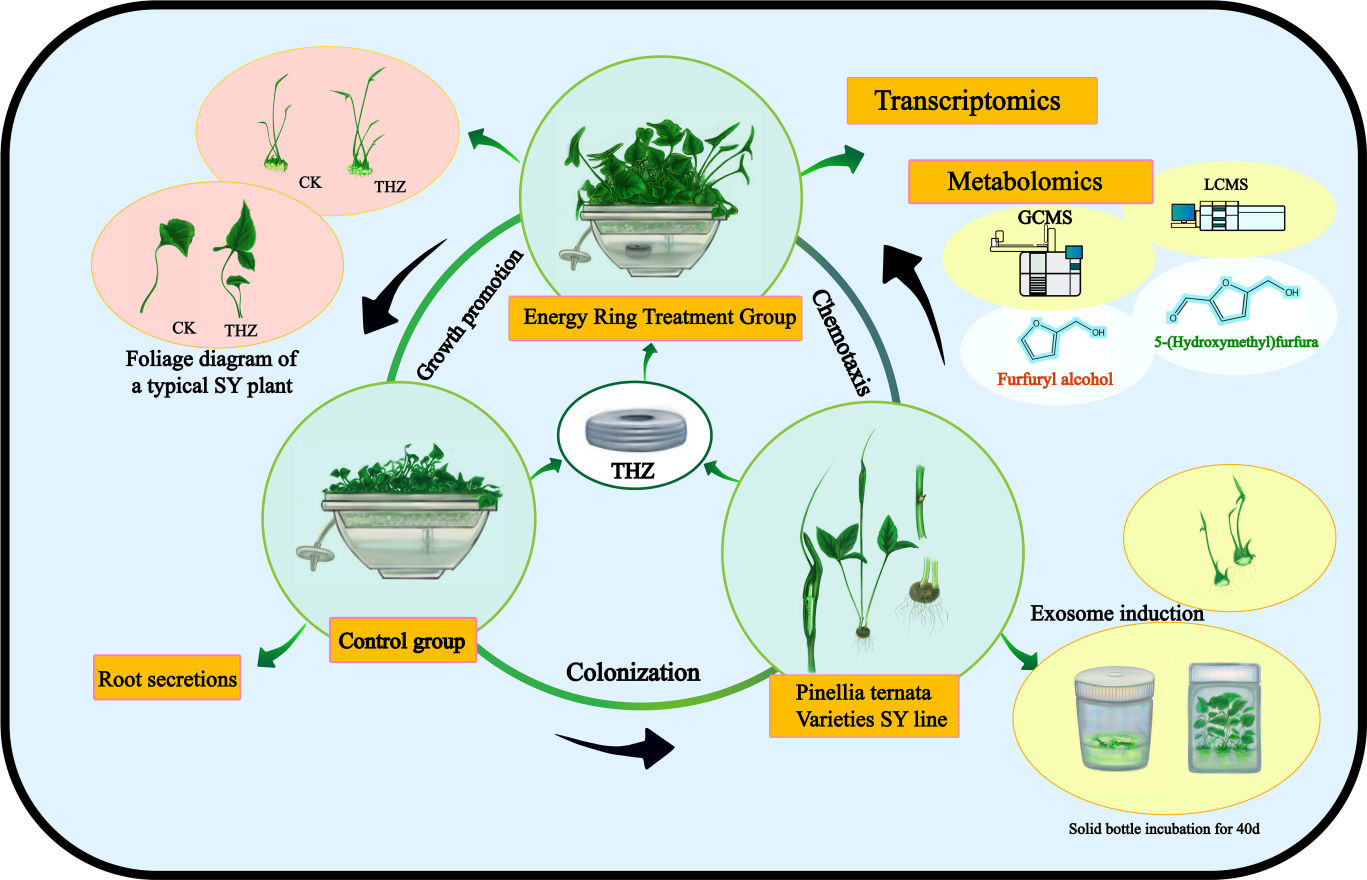
Schematic diagram of growth interactions of THZ *of P. ternata*.

Through transcriptomic and metabolomic analyses, we sought to characterize the global response of *P. ternata* to THZ. Analyses were conducted on the genes and metabolites associated with the differential analysis of phytohormone signaling, phytopathogenic interactions, the plant MAPK pathway, and amino acid metabolic pathways. This research sheds new light on the molecular regulatory mechanisms promoting the growth of *P. ternata* (**Figure 1**).

## 2 Materials and methods

### 2.1 Plant materials, plant growth condition, and root exudation determination

#### 2.1.1 Evaluation of morphological characteristics

*P. ternata*, a Guizhou-native peony leaf type from Zunyi city, was denoted as SY. Seedling propagation was done using a temporary immersion bioreactor system (TIBS) of *P. ternate* (Le et al., 2021; Zhang et al., 2022). As explants, 0.5–0.8-cm^3^ cluster buds were selected and transferred to a liquid medium containing 500 mL of sterile proliferated cluster bud (MS medium + 6-BA 1.0 mg/L + NAA 0.02 mg/L + sucrose 30 g/L, pH 5.8–5.9). The culture medium was observed for 3 days and was confirmed to be contamination-free. The liquid medium was transferred for the multiplication of clustered bud clusters to TIBS. The inoculum of aggregated seedlings is 40 pieces/L, and the frequency of intermittent immersion is 5 min every 12 h. Then, they were cultured in a tissue culture room at (25 ± 1)°C with a light intensity of 2000 Lx–3000 Lx and a light cycle of 16 h per day, for the phenotypic analysis of *P. ternata.* Under THZ energy-ring stress, the growth of TIBS-grown plantlets was monitored for 60 days. The leaf lengths and widths, petioles, and plant heights of 30 plantlets were determined. The fresh and dried weights and biomass of 30 plantlets were determined, as well as their length/width ratios, proliferation coefficients, and dry matter rates.

#### 2.1.2 Extraction of root exudate

60-day-old, sterile *P. ternata* seedlings were chosen and thoroughly rinsed three times with deionized water. THZ is the experimental group, whereas CK is the control group. Sterilized *P. ternata* seedlings were grown in the dark for 1 day. 100 mL of liquid was filtered through a 0.22-micron filter membrane and shaken for 6 h in a greenhouse. The liquid was put in the rotary evaporator, after which 50 mL was added, and then 100 mL of dichloromethane was added. It was shaken for 8 h. Then, the mixture was filtered through a 0.45-micron filter membrane. The mixture was stored at 4°C in the refrigerator for GC-MS analysis (Han et al., 2022).

#### 2.1.3 GC-MS analysis of root exudates

##### 2.1.3.1 Measurement conditions

The temperature was maintained for 3 min at 280°C for the injection and 50°C for the column. Using a 10°C min^−1^ program, the temperature was increased to 290°C and maintained for 20 min; it served as the carrier gas. The injection volume and flow rate are 1 mL/min and lu/mL, respectively. The MS electron bombardment source is 70 eV. The entire operation has a scan speed of 0.2 s. The interface temperature is 250°C, whereas the ion source is 200°C. Computer retrieval was utilized to ascertain unfamiliar compounds and their relative contents (Mohan et al., 2020; Lopez-Guerrero et al., 2022).

### 2.2 Transcriptome analysis

#### 2.2.1 Sequencing experiment procedure

The Illumina NovaSeq 6000 sequencing technology provides the foundation for eukaryotic mRNA sequencing, which sequences all the mRNA produced in a given time period by certain eukaryotic tissues or cells (Shaw et al., 2021). The Illumina TruSeq™ RNA Sample Pre-Kit is used in the sequencing experiment to build a library. The workflow and instrument reagents are as follows.

#### 2.2.2 Total RNA extraction

We extracted RNA from each *P. ternata* leaf to create a single database using RNA extraction kits (TSINGKE and TSP401). We assessed the concentration and purity of the isolated RNA using NanoDrop 2000 (Fu et al., 2021).

### 2.3 Metabolome analysis

The samples used for the metabolomics assay were consistent with the transcriptome, both being *P. ternata* leaves. Using an ACQUITY UPLC ® HSS T3 (2.1 150 mm, 1.8 m) (Waters, Milford, MA, USA) chromatographic column, the Thermo Vanquish (Thermo Fisher Scientific, USA) ultra-high performance liquid phase system operates at a flow rate of 0.25 mL/min, a column temperature of 40°C, and an injection volume of 2 µL (Liu et al., 2020b; Fu et al., 2021).

### 2.4 Validation of qRT-PCR

Both qRT-PCR and transcriptome sequencing were used to employ the same RNA. Total RNA was processed using DNase, transformed to cDNA for qRT-PCR, and then reverse-transcribed using the PrimeScript RT reagent kit (TAKARA, Dalian, China). The ACAD gene was used as a reference to normalize the relative expression levels (Choi, 2020). qRT-PCR was performed using qTOWER real-time thermal cyclers from Analytik Jena in Germany. The relative expression rate was calculated using the 2^−ΔΔCt^ technique (Hao et al., 2020). A total of 11 genes, including up- and downregulated genes, were chosen at random. The validation gene primers are included in **Table S1**. Simple linear regression analysis was used to link the results from qRT-PCR with the transcriptome (Sun et al., 2021).

### 2.5 Statistical analysis

We used a statistical tool called SPSS 22.0 to analyze the data and performed at least three biological replicates for each analysis. The mean and SD of the results and errors were calculated from these replicates. A P-value of less than 0.05 was deemed statistically significant.

## 3 Results

### 3.1 Phenotypic analysis of the experimental and control groups

**Figure 2:**
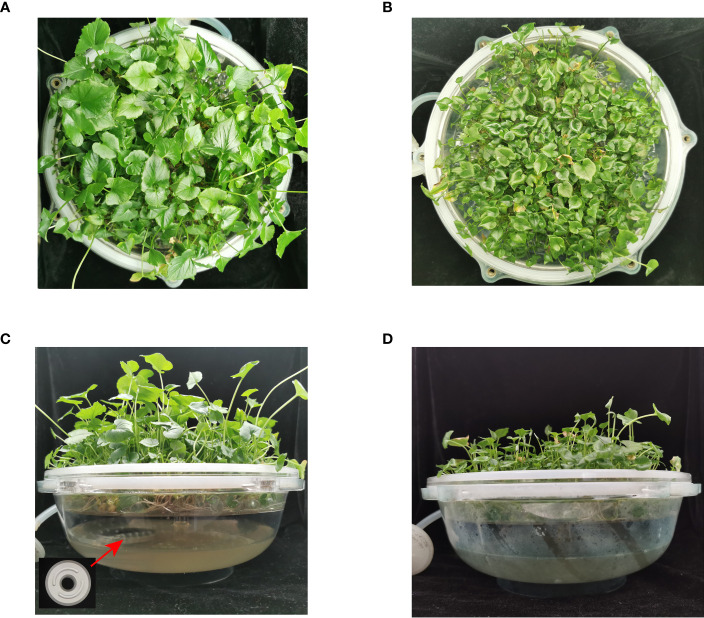
Plots of the effect of THZ growth promotion on TIBS *Pinellia ternata* SY clump seedlings. **(A, C)**: experimental groups; **(B, D)**: control groups.

**Figure 3:**
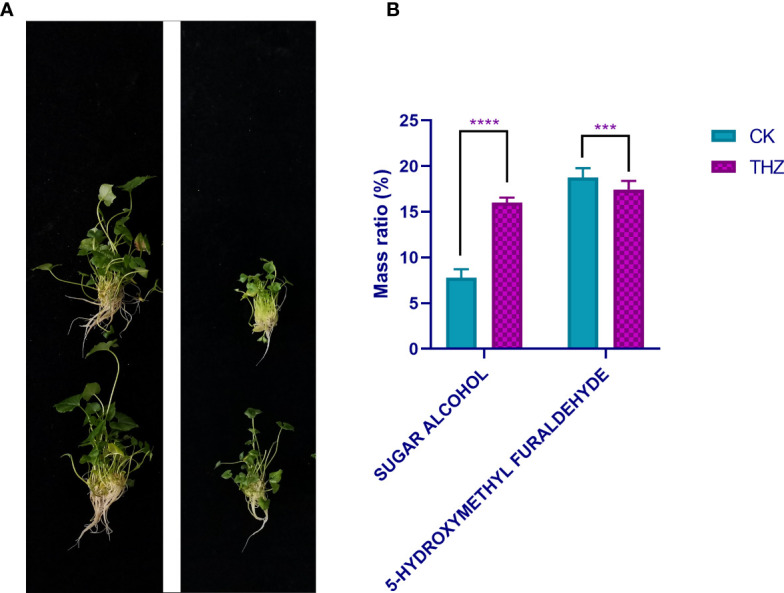
**(A)** Plot of single *P. ternata* clumped seedlings grown in terahertz energy rings for 60 days. **(B)** Analysis of root exudates at CK and THZ treatment levels in *Pinellia ternata.* *** indicates a significant difference (P < 0.01). **** indicates a significant difference (P < 0.001).

Significant differences were found in the THZ group’s petiole length, diameter, and diameter, which were each 125.08% and 56.94% higher than those of the control group, respectively (**Table 1**). The THZ group’s leaf blade length, width, and number of single clump buds were each 120.92%, 147.63%, and 30.05% higher than those of the control group, respectively (**Table 1**; **Figure 2**). Among them, there were no significant differences in root length, but there were extremely significant variances in leaf blade length, breadth, and petiole length (**Figures 3A**, **S1**).

**Table 1:** Growth characteristics of experimental and control groups grown for 60 days in SY *Pinellia ternata*.

Group	Petiole length (mm)	Petiole diameter (mm)	Blade length (mm)	Blade width (mm)	Number of shoots in a single clump (pcs)	Root length (mm)	Fresh weight (g)	Dry weight (g)
**CK**	43.18 ± 2.343b	0.72 ± 0.035b	10.99 ± 0.444b	10.37 ± 0.503b	18.63 ± 1.911b	51.20 ± 1.880a	1.88 ± 0.093b	0.43 ± 0.059b
**THZ**	97.19 ± 3.263a	1.13 ± 0.032a	24.28 ± 0.714a	25.68 ± 1.172a	24.23 ± 1.767a	54.69 ± 1.253a	4.38 ± 0.104a	1.72 ± 0.097a

mean ± standard error; different lowercase letters indicate significant differences (P ≤ 0.05).

### 3.2 Effect of THZ on the root secretion of *Pinellia ternata*

Sugar alcohols work well as complexing agents for vitamins and minerals, including trace elements. They can build stable complexes by combining different nutrients. *Pinellia ternata* root secretion includes 5-hydroxymethyl furfural and sugar alcohols. Compared with CK, THZ had a sugar alcohol percentage that was 108.95% greater. The experiment revealed a highly significant difference in the sugar alcohol content of THZ and CK, which was an increase of 108.95% (**Figure 3B**).

### 3.3 A global overview of the leaf transcriptome of *Pinellia ternata* with THZ

The Illumina NovaSeq 6000 sequenced 130,300,384 clean reads for the control group, whereas the experimental group had 138,509,108 clear reads. In total, 39.38 GB of clean data was collected. Each sample’s clean data surpassed the minimum sequencing quality standards with over 6.11 GB and a Q30 base percentage of 94.3% or higher. **Table 2** shows that over 94.3% of the bases were Q30, essential for proper sequencing.

**Table 2:** Sequencing data statistics of SY *Pinellia ternata* experimental and control groups.

Sample	Clean reads	Clean bases	Q20 (%)	Q30 (%)	GC (%)
CK1	43,366,066	6,332,668,200	98.26	94.79	52
CK2	42,967,620	6,105,785,439	98.24	94.8	50.25
CK3	43,966,698	6,462,748,489	98.26	94.76	52.58
THZ1	49,339,588	7,284,401,019	98.1	94.3	53.01
THZ2	45,682,292	6,719,250,578	98.25	94.74	52.66
THZ3	43,487,228	6,479,021,731	98.18	94.54	53.26

#### 3.3.1 Transcripts and gene lengths of *Pinellia ternate* transcripts

**Figure 4:**
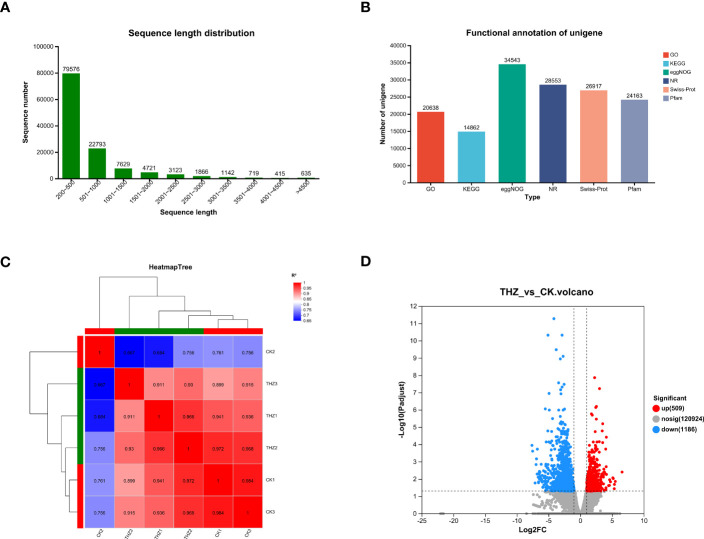
**(A)** Length of transcripts and genes *of P. ternata.* **(B)** Unigene annotated summary tables in various databases. **(C)** Heat map of correlations between samples of the treatment and control groups of *P. ternata.* **(D)** Analysis of genetic significance of differences between the treatment and control groups of *P. ternata.* Volcano plot.

According to the findings, there were 122,619 unigenes and 235,523 transcripts constructed and the average N50 length for each was 1,026 bp and 1,175 bp, respectively. Both 1,026 bp and 1,175 bp were the average N50 lengths. The average base length was 200 bp–500 bp, and there were 79,576 genes, reaching 65% and making up the majority of the population; the average base length was 501 bp–1,000 bp, and there were 22,793 genes, making up 19%; and the average base length was 1,001 bp–1,500 bp, and there were 7,629 genes, reaching 6% and making up a smaller population (**Figure 4A**).

#### 3.3.2 Functional annotation of unigenes

All genes and transcripts derived from the transcriptome assembly were compared using BLAST to 120,378 unigenes from six databases (NR, Swiss-Prot, Pfam, eggNOG, GO, and KEGG). Of the total number of genes identified in the NR database, 28,553 unigenes (23.72%) contained homologous protein sequences. The smallest number of genes in the KEGG database that were annotated was 14,862, or 12.35% of all unigenes. The eggNOG database has the most genes with annotations, 34,543, or 28.7% of the total unigenes. In all databases, 39,584 (32.88%) unigenes could be annotated (see **Figure 4B**).

#### 3.3.3 Heat map of correlation between samples

RNA-seq sequencing was used to analyze the transcriptome of *P. ternata* clumped seedling stages cocultured with THZ energy rings and terminal buds in TIBS—three duplicate biological samples from various treatment groups clustered together based on FPKM value analysis. The biological replicates had reasonably good Pearson correlation values, and samples from various treatments could be discriminated from one another with ease. The inter-sample correlation study showed that further biological investigation might be conducted using the current sequencing data in **Figure 4C**.

#### 3.3.4 Identification and annotation of differentially expressed genes in *Pinellia ternata*

We found 1,695 differentially expressed genes in total, of which 509 showed upregulation and 1,186 showed downregulation, as shown in **Figure 4D**. We found that THZ affected the gene expression in *P. ternata.*

**Figure 5:**
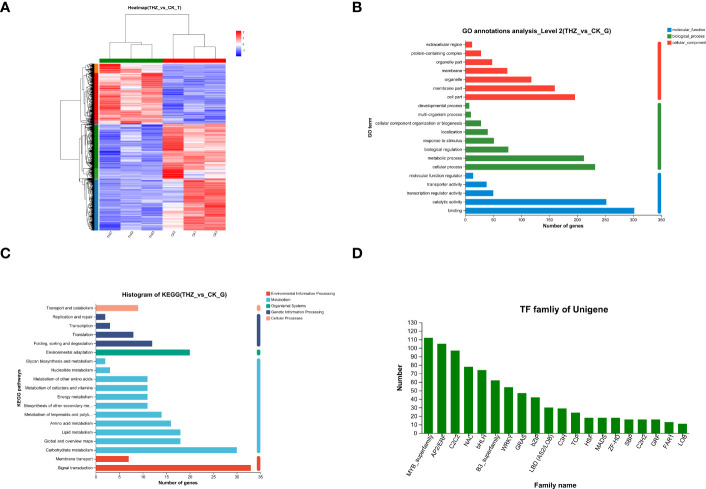
**(A)** Heat map clustering analysis of differentially expressed genes. **(B)** Histogram of GO functional annotation analysis of differential genes. **(C)** Histogram of functional annotation analysis of the differential gene KEGG. **(D)** Statistical chart of transcription factor families.

According to the findings, 1,695 genes showed a differential expression between the THZ-treated 60-day and control groups, indicating that numerous genes were differently expressed under THZ growth conditions. The three samples from the average growth group and the three samples from the THZ treatment were divided, and their expression clustering patterns were different, according to a heat map clustering analysis of all the differentially expressed genes (see **Figure 5A**).

The functional annotation of the differentially expressed genes in *P. ternata* was examined using GO functional pathways, to present the top 20 GO functional annotations in the Biological Process (BP) status and to show steady growth and THZ groupings. Biological process (BP), cellular component (CC), and molecular function (MF), involving eight, seven, and five functional groupings, respectively, were the three main categories in which the genes fell. The findings revealed considerably distinct gene expression patterns for *P. ternata* and cellular and bioregulatory activities and biological processes (see **Figure 5B**).

KEGG annotations were used to examine the differences in metabolic pathways between the control and treated groups, *P* ≤ 0.05, and revealed phytohormone signaling, MAPK signaling pathway, and plant–pathogen interaction pathway, indicating that processing of these metabolic pathways may be relevant to the growth of *Pinellia* (**Figure 5C**).

**Figure 6:**
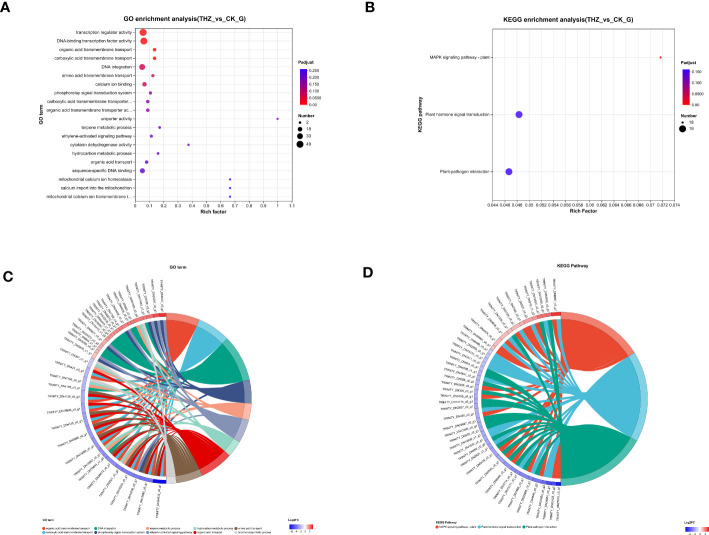
**(A)** Graph of GO enrichment analysis results. **(B)** KEGG enrichment analysis results graph. **(C)** GO-enriched string diagram. **(D)** KEGG-enriched string diagram.

Through GO enrichment analysis, we learned that GO is enriched for transcriptional regulator activity and DNA-binding transcription factor activity in the MF branch and for organic acid transmembrane transport and carboxylic acid transmembrane transport in the BP branch, respectively (**Figure 6A**). The top 115 GO terms associated with the greatest gene enrichment for *P. ternata* growth variations are displayed in the figure. The 115 GO terms enrich the function of differentially expressed genes, including 67 enriched GO biological processes and 42 enriched GO molecular function terms. The GO terms ranked in the top two with transcription regulator activity and DNA-binding transcription factor activity (see **Figure 6C**).

#### 3.3.5 KEGG enrichment analysis results

Plant–pathogen interaction, the plant MAPK signaling pathway, and plant hormone signal transduction are the three areas where KEGG is most enriched, hinting that these pathways could be active in response to abiotic terahertz. The findings imply that these pathways could be relevant in *P. ternata* response to terahertz abiotic stress (**Figure 6B**). To better comprehend *P. ternata* reaction processes, transcriptome sequencing was utilized to characterize both the qualitative and quantitative aspects of gene expression in tissues impacted by THZ. The top 56 KEGG keywords for the most notable gene enrichment for growth variations in *P. ternata* are depicted in the image (**Figure 6D**).

### 3.4 Plant hormone signal transduction pathway and MAPK signaling pathway

**Figure 7:**
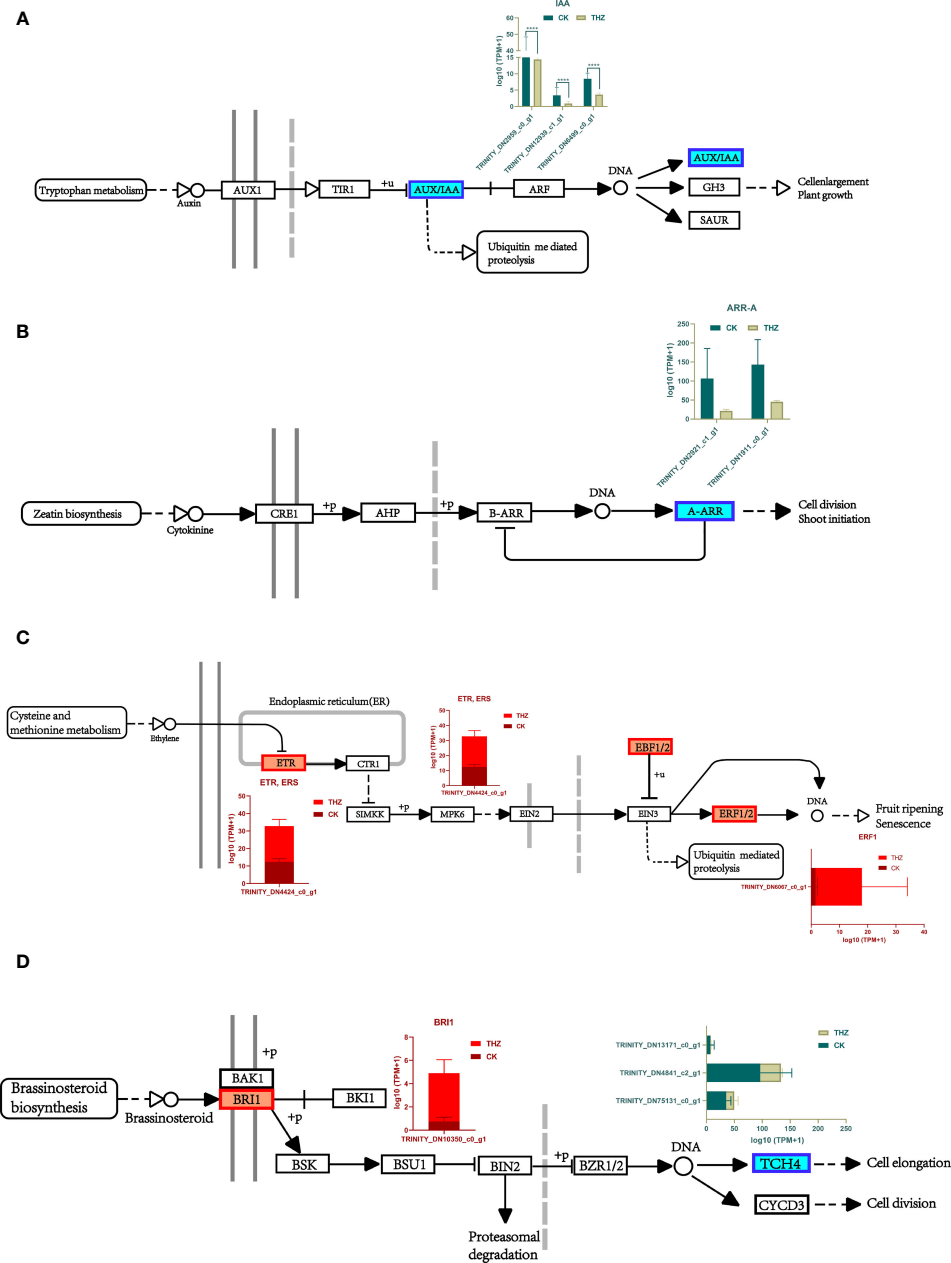
**(A)** Analysis of differential gene expression in the *P. ternata* growth pathway. **(B)** Analysis of differential gene expression in the cytokinin pathway. **(C)** Analysis of differential gene expression in the ethylene pathway. **(D)** Differential gene expression analysis of the rapeseed in lactone pathway. Asterisks indicate significant differences; ****P<0.0001.

A downregulation was observed in the center and downstream of the growth hormone/indole acetic acid-inducible gene (AUX/IAA) of the two modules. More genetic variations in this module might indicate that THZ sped up *P. ternata* growth (**Figure 7A**). The immediate impact of THZ on hemlock development throughout the growth period and the early downregulation of cytokinins may have contributed to the downregulation of A (A- ARR). THZ could have helped *P. ternata* mature early (**Figure 7B**).

Our research shows that this is likely because THZ influences ethylene production in hemlock to promote sprouting and break since the ethylene receptor (ETR) and the ethylene cell signaling gene *EBF1/2* are primarily elevated modules of differential genes implicated in the ethylene signaling pathway. This behavior could have been caused by THZ’s behavior on *P. ternata* ethylene production (**Figure 7C**).

According to the findings, *BRI1* was differentially elevated upstream of *P. ternata* metabolism and downregulated downstream of TCH4, indicating that the THZ energy loop may speed up *P. ternata* metabolism of oleuropein lactone and hence encourage plant development (**Figure 7D**).

**Figure 8:**
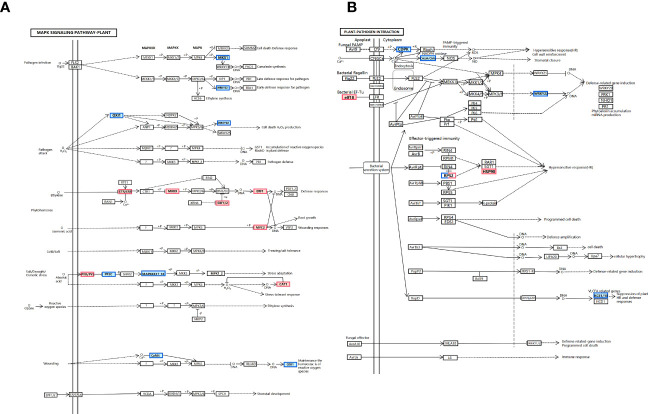
**(A)** MAPK signaling pathway: plant pathway. **(B)** Plant–pathogen interaction pathway.

We find that THZ impacts *P. ternata* growth metabolism (see **Figure 8A**). Additionally, as shown in **Figure 8B**, we discovered that *(CDPK)* and *(WRKY22)* were downregulated in response to THZ waves.

### 3.5 Differential analysis of transcription factors

There were 940 transcription factors from 34 distinct TF families discovered using transcriptome sequencing of *P. ternate* samples under normal and THZ conditions and TF prediction analysis of the assembled unigene. The MYB family has the greatest percentage, followed by the AP2/ERF, C2H2, NAC, bHLH, and B3_superfamily families. Further evidence shows that plants share the MYB and AP2/ERF transcription factor superfamilies. In addition, it was discovered that *P. ternata* have an increased expression of the MYB and bHLH families, which are well-recognized for their resistance in plants. Observing Breit. samples under THZ stress suggests that these transcription factors have a beneficial regulatory function, indicating that transcription factors can react to THZ stress through various regulatory pathways; other transcription factors, including TRINITY_DN30208_c0_g1, a member of the NAC family, were downregulated, as shown in **Figure 5D**.

### 3.6 Metabolic analysis of THZ on the growth of *Pinellia ternata*

**Figure 9:**
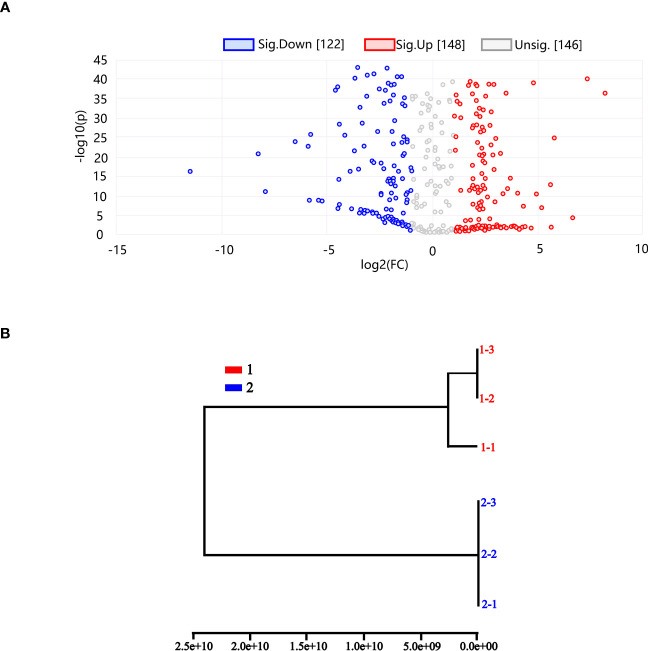
**(A)** Differential metabolite volcano map. **(B)** HCA (hierarchical-clustering analysis) dendrogram. A PCA dendrogram (6PCs). 1 Control group of *P. ternata.* 2 THZ Experimental group *of P. ternata.* Each point in the graph represents a metabolite. The horizontal coordinates represent the log2 value of the multiple of the quantitative difference between the two samples for a metabolite; the vertical coordinates indicate the log10 value of the P value. The higher the absolute value of the horizontal coordinate, the more significant the difference in expression multiplicity between the two samples for a metabolite; the larger the vertical coordinate value, the more significant the differential expression and the more reliable the differentially expressed metabolites obtained from the screening. The red points in the graph represent an upregulated differential expression. The red dots represent upregulated differentially expressed metabolites, and the blue dots represent downregulated metabolites. The gray dots represent metabolites detected but did not meet the filtering parameters.

Using differential metabolite volcano plots, 416 metabolites in total (148 upregulated and 122 downregulated) were examined, as shown in **Figure 9A**. The same subgroup was generated in controls 1–3 and 1–2, and the two subgroups belonged to the same group, according to PCA-based HCA dendrogram analysis. We find that the other groups had both quantitative and qualitative differences. The fact that treatment groups 2–1, 2–2, and 2–3 belonged to the same subgroup shows that the quantitative and qualitative metabolite alterations for the THZ-treated metabolites were more significant than for the other samples in **Figure 9B**.

**Figure 10:**
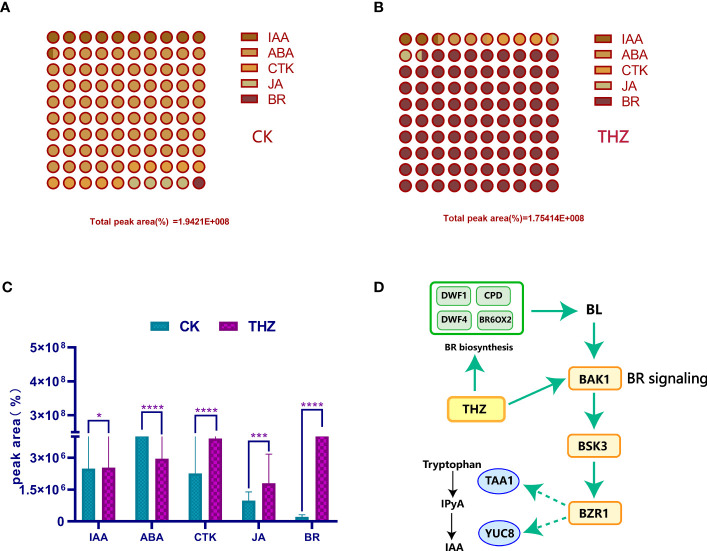
**(A)** Percentage of peak area of phytohormones in the control group. **(B)** Percentage of peak area of phytohormones in the THZ group. **(C)** Significant difference analysis of phytohormones in the experimental group THZ and control group CK. **(D)** Pathway of the effect of THZ on BR and IAA in of *P. ternata*. The indicated statistical differences are calculated using the one-way ANOVA and Bonferroni’s *post-hoc* test: *P < 0.05; ***P < 0.001; ****P < 0.0001.

By analyzing **Figure 10C**, we discovered that the THZ group had significantly higher levels of brassinolide (BR), cytokinin (CTK), and jasmonic acid (JA) compared with the CK group. Brassinolide promotes healthy growth, flower bud differentiation, and plant growth and development. Additionally, it boosts the growth of *P. ternata* cells, accelerates sugar breakdown, and facilitates water uptake in *P. ternata*.

### 3.7 Integrative analysis between the transcriptome and metabolome

To better comprehend the relationship between DEGs and SRMs, the transcriptome, and the metabolome, we performed a combinatorial analysis of both the transcriptome and the metabolome. The metabolome and transcriptome both contain pathways significantly enriched in phytohormone metabolism.

The figure depicts *P. ternata* metabolites. **Figure 10** shows that the control group had the greatest proportion of CTK and IAA, whereas **Figure 10B** shows that the THZ-treated group had the highest percentage of BR and CTK. In **Figure 10C**, the THZ group had a much larger proportion of BR, CTK, and JA than the CK group. The abiotic stress caused by THZ had an impact on BR biosynthesis, activating BAK1, BSK3, BZR1, and YUC8TAA1 and affecting tryptophan, which in turn activated IPyA and IAA (**Figure 10D**).

**Figure 11:**
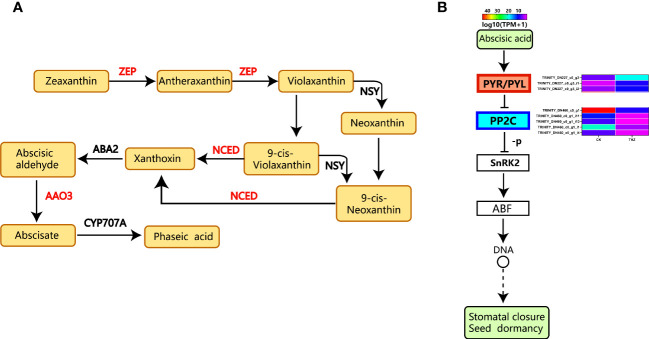
**(A)** ABA biosynthetic pathway (Taylor et al., 2000). **(B)** Analysis of differential gene expression in the abscisic acid pathway.

Zeaxanthin to antheraxanthin to violaxanthin to neoxanthin or 9-cis-violaxanthin to xanthoxin is the first step in the biosynthesis of ABA—phaseic acid from abscisic aldehyde to abscisate (**Figure 11A**) (Taylor et al., 2000). The most well-known family of hormone receptors in plants is *PYR/PYL*. The abscisic acid signaling process increased the upregulation of certain *PYR/PYL* differential genes (Ng et al., 2014). For instance, TRINITY_DN227_c0_g3, TRINITY_DN227_c0_g3_i1, and TRINITY_DN227_c0_g3_i2 are the gene codes. Protein phosphatase (PP2C) may have been downregulated due to the THZ energy loop’s impact on *P. ternata* proliferation. As shoots emerge from dormancy, the expression of the genes controlling shoot dormancy decreases. As a result, there was substantial differential gene expression in the abscisic acid signaling pathway (**Figure 11B**) (Wang et al., 2019).

### 3.8 qRT-PCR validation

**Figure 12:**
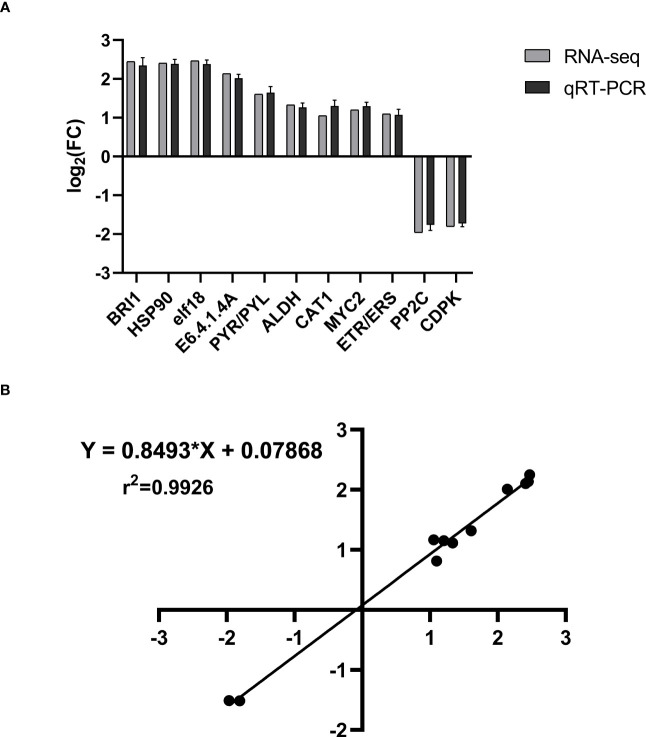
The validation of transcriptome using qRT-PCR. **(A)** The 11 genes’ expression pattern of transcriptome and qRT-PCR. The columns in black and gray denote the expression value of transcriptome and qRT-PCR, respectively. The value represents the log2 fold change in the THZ group compared with the control group. **(B)** Correlation of transcriptome (x-axis) and qRT-PCR (y-axis) data. Values are means of three biological replicates—error bars indicate ± SE. Data are presented as means ± SE (n = 3). T-tests determined statistical significance.

We chose 11 genes at random for qRT-PCR experiments to verify the DEGs’ expression pattern. 18sRNA was used as the internal reference gene. These genes comprised up- and downregulated ones, as well as ones with high and low manifestation rates. The 11 genes’ primary expression patterns were similar to those shown in **Figure 12A**. With a Pearson r-value of 0.9965, the correlation between the transcriptome and qRT-PCR was quite positive. The R-squared value for goodness of fit was 0.9926 after the linear regression for the correlation study was completed (**Figure 12B**). These findings demonstrated the reliability of the transcriptome.

## 4 Discussion and perspectives

It has been shown that the THZ energy loop enhances the elevation of plant root secretions. We demonstrate that the phytohormones growth hormone, cytokinin, ethylene, abscisic acid, oleuropein lactone, and jasmonate can all be controlled by THZ. Growth hormones are stimulated in the rhizosphere during plant growth (Mu et al., 2022). Furthermore, several researchers have demonstrated that inter-root bacteria promote the growth of rhizobia (Zhou et al., 2023). Pseudomonas populations are stimulated by bacterial growth in native soils to improve plant disease control (Tao et al., 2020; Wang et al., 2021a). Here, we concentrated on THZ and plant growth interactions.

The biology of phytoplasma and its presence are still unclear because of the peculiar lifestyle of phytoplasma and the difficulties in keeping them *in vitro* for performing controlled infections (Kirdat et al., 2023). However, much fresh information has recently become available, supported by new transcriptome and metabolomic data from host plants infected with phytoplasma.

Recent discoveries of several *Arabidopsis* and other plant hormone transporters have shed light on the active mechanisms governing spatiotemporal hormone control (Park et al., 2017; Anfang and Shani, 2021; Chae et al., 2022). The ability to identify additional hormone metabolites (precursors, catabolites, and conjugates) in a single sample, thanks to recent technological advancements in analytical approaches, has allowed researchers to learn more about the overall structure of the hormone metabolome (Isoda et al., 2021). A sufficient extraction solvent is essential to prevent enzymatic degradation, reduce the concentrations of interfering compounds, and effectively extract analytes from plant tissues (da Silva and Magalhaes, 2023). Typically, ACN extraction is used in non-targeted metabolomic studies (Yu et al., 2023a). Chemical derivatization is essential for gas chromatography but not LC-MS-based multitargeted profiling since most known phytohormones are non-volatile substances (Nwogha et al., 2023; Zhang et al., 2023). The versatility of LC methods has been significantly enhanced with the growing availability of chromatographic columns with various physicochemical features. Because of this, LC is now the most trustworthy, useful, and widely applied method for simultaneous phytohormone analysis (Delatorre et al., 2017; Blatt-Janmaat et al., 2023; Del Mondo et al., 2023; Joshi et al., 2023; Xu and Xiong, 2023).

THZ can start and activate several important plant processes, including growth, maturation, and primary metabolism (Wu et al., 2023; Yu et al., 2023b). However, it can also impact several plant processes, including the buildup of secondary metabolites. THZ may interact with various environmental natural resources, particularly plants (Nourinovin et al., 2023). The roots of the plants are in direct contact with these THZ in the rhizosphere (Zhao et al., 2023).

Plants regulate the anions and cations in their cells to maintain an electroneutral environment, a balanced ion composition, and metabolic activity. Abiotic stress, however, swiftly upsets this balance (Saito and Uozumi, 2020; Wu et al., 2021; Zhang et al., 2022). For instance, P significantly reduced the bioavailability of uranium (U) and U accumulation in Arabidopsis thaliana in prior research, lowering U detrimental effects on root cells and development (Cui et al., 2022; Laanen et al., 2023).

Based on the research’s conclusions, it is important to note THZ wave interactions’ role in the biological control of plant development. We demonstrate that altering water molecule form impacts regulatory genes in plant roots, stems, and leaves (Yamazaki et al., 2020). Plant growth is aided by signaling pathways, which are particularly rich in phytohormones (Salvi et al., 2021).

Our study is the first to suggest a mechanism by which THZ responds to the growth promotion of *P. ternata*. THZ can change the hydrogen bonding structure of water molecules from a polymeric macromolecular structure to a small molecular structure arranged in straight chains (Elgabarty et al., 2020; Zhao et al., 2020). It is speculated that its frequency is exactly the resonant frequency of electromagnetic waves at which plant growth undergoes bioelectronic communication, so that terahertz water can promote plant growth (Hassanien et al., 2014; Vian et al., 2016).

Analysis of the transcriptomic results showed that *BRI1* gene expression is significantly high in the phytohormone pathway and that *BRI1* acts as a heterodimer controlling the production of BR and subsequent growth responses (Liu et al., 2020a). *BRI1* acts in most plants to promote growth, and brassinosteroids (BRs) are important plant hormones that promote cell elongation and division (Mao and Li, 2020). Studies in *poplar*, the model woody plant species, have shown that elevated levels of BR promote secondary growth and that overexpression or mutation of *BRI1*, the receptor for BR signaling, leads to changes in poplar growth and wood development (Benitez-Alfonso and Caño-Delgado, 2023). In the metabolites, our findings showed that BR was significantly increased. In the present study, several DEGs were annotated to the phytohormone pathway. However, annotating these genes suggests a role for these genes in the *P. ternata* growth pathway; we still need to determine the exact role encoded by these genes. There were 19 differentially expressed genes in the KEGG-enriched phytohormone signaling pathway, 13 expressing downregulation and six expressing upregulation, suggesting their potential role in THZ on dipterocarp growth (Altmann et al., 2020). There were 11 DEGs randomly selected for validation by qRT-PCR. All genes were identical between the sequencing data and the qRT-PCR method (see **Figure 12**).

## 5 Conclusions

In this study, the growth potential of *P. ternata* was evaluated. Under THZ stress, *P. ternata* strains performed better than other strains. Regarding growth potential and antioxidant capacity, THZ-stressed varieties beat the control group. Numerous genes and metabolites associated with phytohormones were found by analyzing the transcriptome and metabolome. Numerous genes and metabolites were shared between the experimental and control groups. However, the THZ-treated group’s higher accumulation and regulatory capacity suggest the likelihood that this is the primary reason underpinning *P. ternata* strong growth potential—the pathway for transducing plant hormone signals is the plant-based MAPK signaling pathway. The plant–pathogen interaction pathway was greatly enriched in the THZ group. DEGs and SRMs were more prevalent in the THZ experimental group than in the control group. These results suggest that the response to *P. ternata* growth depends on the plant hormone signal. The THZ-treated group advances our knowledge of the regulatory mechanisms underlying the THZ response in *P. ternata.*

## Data availability statement

The original contributions presented in the study are publicly available. This data can be found at NCBI with the accession number PRJNA983723.

## Author contributions

DW wrote the first draft and composed the figures and formal analysis. CZ contributed to editing. All authors critically reviewed and commented on the manuscript. All authors contributed to the article and approved the submitted version.
