# Supplementary material for: Analysis of the response mechanisms of Pinellia ternata to terahertz wave stresses using transcriptome and metabolic data
Source: Front Plant Sci. 2023 Sep 12;14:1227507. doi: 10.3389/fpls.2023.1227507 (PMC10522861; doi:10.3389/fpls.2023.1227507)
Supplement: Supplementary file 1 [file DataSheet_1.docx]

**Supplementary Table S1:Primers used in this study**

| **Primer** | **Sequence (5′→3′)** | **Amplification length (bp)** | **Tm (°C)** |
| --- | --- | --- | --- |
| ***18SRNA*** | CGCATATAAATAAACGGAGGAA | （151bp） | 61℃ |
|  | GACGCTTCTACAGACTACA |  | 60℃ |
| ***PtETR/ERS*** | GCCACGGCCTACTTCTCGATTCCC | （156bp） | 66℃ |
|  | CGAGTGTGCCTCGTAGGTGAACAC |  | 62℃ |
| ***PtPYR/PYL*** | TCCACTCTCCCCAGGTCCTCCCTC | （91bp） | 66℃ |
|  | GGTTAGCGGTACTGCCGCAGCTGA |  | 66℃ |
| ***PtCAT1*** | GAGAACGAGCAGCTCGCCTTCTCG | （153bp） | 65℃ |
|  | GGGGGCGTTCACAGGCAGCATCAG |  | 68℃ |
| ***PtBRI1*** | GCGGCGTCCGGGAGCTCTATCTTG | （121bp） | 67℃ |
|  | GGGGCGAGGGTAGAAAGGGTGTTG |  | 65℃ |
| ***PtMYC2*** | TCCTCCGCCTACTTCAACCAGGAG | （112bp） | 63℃ |
|  | CCACATCCACCGACGACTGCCAAA |  | 65℃ |
| ***PtPP2C*** | AGGGGTCCCTGTCCCCCTCTCATC | （211bp） | 67℃ |
|  | ACCAGATGTTAGCAGCGGTGGCGC |  | 68℃ |
| ***PtHSP90*** | CCCTCTTGATACGGTTGCCGAAGT | （105bp） | 64℃ |
|  | AATGCACATTGATGGCCACCCTCC |  | 63℃ |
| ***PtCDPK*** | GAGCTGTTAGCCGCTTTGTAGGAT | （140bp） | 62℃ |
|  | AAGTGAACAAGGAATTGCCCAAGC |  | 61℃ |
| ***Ptelf18*** | CCTCTTCTCCGCCTCCCCACACGG | （164bp） | 68℃ |
|  | GCTGCTTGTCGCCATGGGGCAGCA |  | 70℃ |
| ***PtE6.4.1.4A*** | CCCAAGCGAAAGCGAAAATCTTCA | （116bp） | 60℃ |
|  | CTCCAGCAGTAAATCTCGCTGCCA |  | 61℃ |
| ***PtALDH*** | AACGTGCTTTGAAGCGCACAGTAG | （153bp） | 62℃ |
|  | CGACTCTACCACCTCCGGACTGGA |  | 63℃ |

**Supplementary Table S1:18SRNA was used as the internal reference gene. Results were analyzed using the 2^-ΔΔCt^ relative quantification method, with three replicates of each sample.**


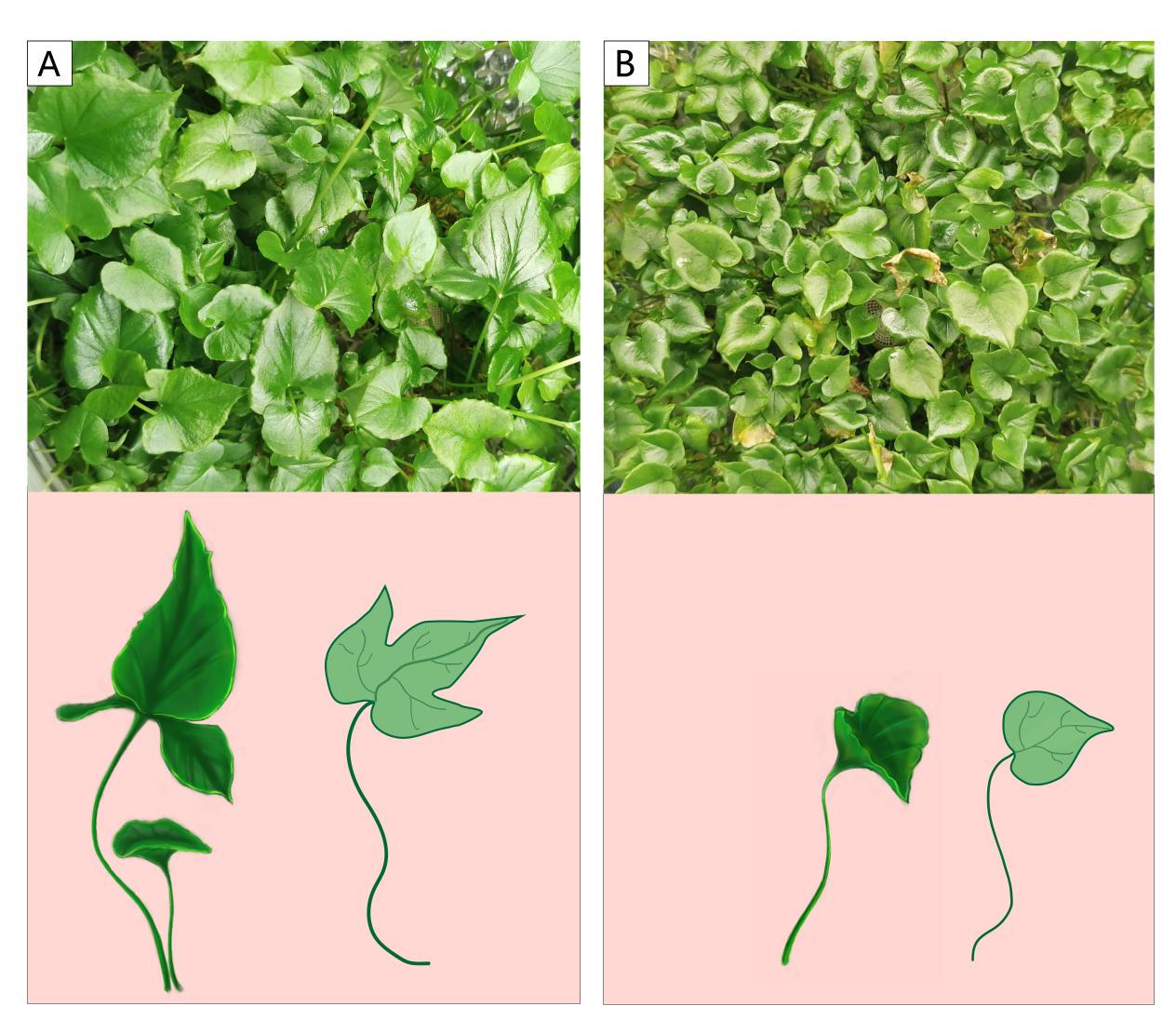


**Supplementary Figure S1. Schematic diagram of the phenotype and orientation of the leaves *of Pinellia ternata.* A, Three-leaf phenotype, with three differently oriented leaves;B, Single-leaf phenotype, one leaf;**

Dongdong Wang, et al., Supplementary Figure S1
